# Unobtrusive Sensing Technologies for the Lifecare Solution

**DOI:** 10.1155/2019/7597190

**Published:** 2019-07-09

**Authors:** Hyun Jae Baek, Ahyoung Choi, Jilong Kuang, Heenam Yoon

**Affiliations:** ^1^Department of Medical and Mechatronics Engineering, Soonchunhyang University, Asan, Republic of Korea; ^2^Department of Software, Gachon University, Seongnam, Republic of Korea; ^3^Samsung Research America (SRA), Mountain View, CA, USA; ^4^Artificial Intelligence Laboratory, Software Center, LG Electronics, Seoul, Republic of Korea

The paradigm for healthcare is not only transforming from hospital-oriented treatments to individually centered prevention but also extending its scope from disease-oriented to wellness-centered. One of the key requirements for care of entire life is to detect health and wellness status accurately and sustainably in various life scenarios [1].

A variety of technologies are currently used to track a person's health and wellness status. They include electrodes, optical sensors, strain gauges, ultrasound devices, etc., each of which has some drawbacks in terms of user experiences such as comfort and convenience or its performance, more specifically, accuracy. The objective of emerging unobtrusive sensing technology is to enable sustainable tracking of physical activities and behaviors, as well as physiological and biochemical parameters during daily life, while ensuring accuracy. This may also include estimating health-related indexes that are difficult to measure unobtrusively by using a combination of various physiological signals measured in a noninvasive and unobtrusive manner.

The person being monitored would not even notice the existence of the sensing device or procedure [2]. Unobtrusive sensing technologies, which can be implemented in the form of wearables and IoT devices, may be a good solution for the future lifecare, but there is a difficulty in deriving useful information from low-quality signals [3, 4].

The goal of this special issue is to share cutting-edge research and applications on unobtrusive sensing solutions such as sensors, devices, and signal processing algorithms. For this, the editorial team focused on the core technologies that could contribute to the implementation of possible future unobtrusive lifecare devices and identified the seven representative manuscripts submitted to the special issue.

This special issue includes 1 review paper and 6 research papers on state-of-the-art health sensing technologies that are being studied and developed for lifecare. In the review article entitled “Current Status and Prospects of Health-Related Sensing Technology in Wearable Devices,” J. Cho investigated the healthcare-sensing functions of current commercially available wrist-wearable devices and their technological limitations and prospects. In the article entitled “Noise-Robust Heart Rate Estimation Algorithm from Photoplethysmography Signal with Low Computational Complexity,” J. Shin and J. Cho applied noise robust oscillator-based adaptive notch filter algorithm to trace the heart rate frequency in low signal-to-noise ratio PPG signal. In the article entitled “Quantitative Assessment of Autonomic Regulation of the Cardiac System,” J. K. Wu et al. described a systematic method for the quantitative assessment of autonomic cardiac system regulation based on homeostasis and probabilistic graphic model using physiological parameters. In the article entitled “An Efficient Deep Learning Approach to Pneumonia Classification in Healthcare,” O. Stephen et al. proposed a convolutional neural network model to extract features from a given chest X-ray image and classify it to determine the presence of pneumonia. In the article entitled “Using Kinect v2 to Control a Laser Visual Cue System to Improve the Mobility during Freezing of Gait in Parkinson's Disease,” A. Amini et al. proposed a new indoor method for casting dynamic and automatic visual cue for improving mobility of people with Parkinson's disease based on skeletal information acquired in real time from a Kinect v2. In the article entitled “Estimation of Breathing Rate with Confidence Interval Using Single-Channel CW Radar,” I. Nejadgholi et al. proposed an algorithm for breathing rate estimation from single-channel continuous wave radar using time-frequency analysis to extract Doppler frequency of the radar signal over time. In the article entitled “Development of a Wireless Health Monitoring System for Measuring Core Body Temperature from the Back of the Body,” Q. Wei et al. explored a wireless semiconductor sensor to measure core body temperature at the skin surface of the back under the neck.
